# Post-pandemic trends in youth sport: structural and social determinants of girls’ and women's engagement in Ultimate

**DOI:** 10.3389/fspor.2026.1958579

**Published:** 2026-09-15

**Authors:** Sarah Stokowski, Chris Corr, Andrew Rudd

**Affiliations:** 1 Department of Educational & Organizational Leadership Development, Clemson University, Clemson, SC, United States; 2 School of Business, Franklin Pierce University, Rindge, NH, United States

**Keywords:** gender disparities, mixed methods, post-pandemic engagement, women and girls, youth sport participation

## Abstract

**Introduction:**

The COVID-19 pandemic disrupted youth sport participation in the United States, with evidence suggesting uneven recovery and persistent gender disparities. This study examined patterns of women's and girls' engagement in Ultimate (commonly referred to as ultimate frisbee) from 2014 to 2024, considering structural and cultural contexts.

**Methods:**

Using a multisource mixedmethods design, we analyzed national participation data, survey responses from 1,892 players, qualitative interviews with 14 former participants and one parent, and a 12-month social-media content analysis of 12,460 mentions.

**Results:**

Descriptive registration analyses showed lower participation among women and girls than boys and men, with differences particularly evident in youth and high school pathways. Interviews described structural barriers, social-evaluation concerns, and perceived misalignment between developmental pathways and inclusion values in relation to disengagement. Social-media analysis identified differences in gender-associated content and visibility, although these data do not establish population-level differences in visibility or causation.

**Discussion:**

Taken together, the findings are consistent with the possibility that structural, cultural, and developmental conditions are associated with participation, but the study design cannot establish causality or determine whether disengagement reflected diminished interest. Practice implications include evaluating strategies to support belonging, safety, skill development, equitable programming, visibility, and leadership opportunities. Interpretation is limited by the voluntary cross-sectional survey, purposive qualitative sample, distinct sampling frames across data sources, and limitations of the registration and social-media data.

## Introduction

Youth sport participation in the United States underwent substantial disruption and transformation in the wake of the COVID-19 pandemic. Although organized sport opportunities have resumed following widespread closures in 2020, emerging evidence suggests that participation patterns did not rebound to pre-pandemic norms in the United States. Instead, the pandemic served to reshape developmental sport pathways, exacerbate structural inequalities, and intensify longstanding gender disparities (1–3). While participation in organized sport partially recovered post-pandemic, national estimates indicate that participatory rates in youth sports remain lower than pre-2020 levels, and recovery has been uneven across socioeconomic and demographic groups (4). Notably, youth athletes from higher-income households and communities with established sport infrastructures returned at higher rates, reinforcing persistent structural barriers to access (5). In addition, gender disparities have been particularly pronounced.

Even prior to the COVID-19 pandemic, girls’ participation in organized sport had declined more sharply than boys’ in early adolescence (6). Pandemic-related disruptions altered young athletes’ opportunities for physical activity and social connection, with implications for sport engagement and re-engagement after restrictions (7, 8). However, research has consistently demonstrated that girls’ disengagement from sport was rarely attributable to diminished interest in physical activity. Instead, dropout reflected interpersonal, cultural, and structural barriers embedded within sport systems themselves (9, 10). These broader participation dynamics raise critical questions for emerging and nontraditional sports.

Ultimate (commonly known as ultimate frisbee) has historically positioned itself as a values-driven sport grounded in inclusion and gender equity (11). Recent participatory trends, however, suggest declining engagement among women and girls, particularly within youth and high school pipelines (12). Understanding whether such a decline reflects waning motivation or structural misalignment is essential for sport managers seeking to build sustainable and inclusive participation sport systems, both within the context of Ultimate and similarly situated nontraditional sports. Accordingly, **t**he purpose of this study was to examine declining participation among women and girls in Ultimate through a comprehensive mixed-methods approach. Drawing on national participation data from 2014 to 2024, qualitative interviews, focus groups, survey responses, and social media analysis, this study investigated the structural, cultural, and developmental factors shaping women's and girls’ engagement and disengagement in a post-pandemic context. By situating Ultimate within broader trends in youth sport participation and gender equity scholarship, this research contributes to sport management literature on participation sustainability, organizational design, and gendered sport structures.

## Literature review

### Youth sport participation post-pandemic

The COVID-19 pandemic produced unprecedented disruption to organized youth sport in the United States. School closures, facility shutdowns, and restrictions on group gatherings resulted in substantial declines in structured sport participation (1, 3). Scholars have increasingly contended that the pandemic did not merely interrupt participation but altered developmental trajectories and widened preexisting disparities (2). Youth sport systems—already characterized by unequal access and escalating costs—have become even more stratified during the recovery period. In fact, national data indicates only partial recovery in organized sport participation, with overall rates remaining below pre-pandemic benchmarks (4). Additionally, reengagement was uneven across socioeconomic groups, with higher-income families demonstrating faster returns to organized activities. Such patterns reinforce longstanding evidence that youth sport participation is shaped more by structural access, institutional support, and resource availability than by individual motivation alone (13).

Adolescence represents a particularly vulnerable period for sport dropout. Transitions into middle school and high school coincide with increasing selectivity, specialization, and performance pressures within sport systems (14). Pandemic-related interruptions during this developmental window were especially consequential. Adolescents reported declines in perceived competence and weakened peer relationships—both central predictors of sustained participation (7, 8). However, youth athletes who did not return to sport following pandemic closures frequently cited logistical and structural barriers (e.g., increased costs, transportation challenges, program availability) rather than a loss of interest (1). Saliently within the context of the present study, girls reported concerns related to social evaluation and psychological safety within competitive sport environments (2).

### Gender disparities in adolescent sport participation

Gender differences in sport participation have been well established across sport types and cultural contexts. Beginning in early adolescence, girls’ participation in organized sport routinely declines more steeply than boys’ (6). Systematic reviews identify multilevel determinants of girls’ sport dropout, including intrapersonal factors (e.g., perceived competence), interpersonal factors (e.g., peer support), and structural factors (e.g., limited access to female-centered programming) (9). Perceived competence and fear of negative evaluation have consistently emerged as central predictors of dropout among adolescent girls in organized sport programs (10, 14).

Messner (15) found that gendered sport cultures prioritizing physical dominance or early specialization further marginalize girls who do not conform to traditional athletic norms. In addition, girls frequently report heightened sensitivity to team climate and social belonging, suggesting that relational dimensions of sport environments are particularly salient for sustained engagement (16). While such individual factors are salient, structural and symbolic factors also shape participation trajectories of adolescent girls in youth sport. Given media coverage and sport promotion historically privileges men's sport and athletes, such privilege both directly and indirectly reinforces hierarchies of visibility and legitimacy among women and girls sport (17). Underrepresentation of women in coaching and leadership positions furthers limits identification opportunities, signaling constrained long-term pathways within sport organizations (18). Collectively, the body of youth sport scholarship underscores girls’ sport dropout as a reflection of systemic patterns rather than individual disinterest.

### Generational context & organized sport

Generation Z and Generation Alpha occupy middle and high school sport environments during the study period, bringing distinct social and developmental contexts as influential factors to participatory patterns. According to extant research, Generation Z adolescents report high levels of social media engagement and heightened awareness of mental health and wellbeing (19). Notably, increased screen time has been associated with lower physical activity levels, sleep disruption, and elevated symptoms of anxiety and depression (20, 21), each of which serves to influence youth sport participation among Generation Z athletes. Among girls specifically, digital environments that amplify appearance-based comparisons tend to intensify body dissatisfaction and self-consciousness, making public sport participation feel more exposing and emotionally *risky* (22).

Generation Alpha—whose early childhood experiences have been shaped by pandemic instability—often perceive organized sport as less routine and more evaluative than previous generational cohorts. Adolescents who have experienced prolonged sport interruptions report declines in confidence and perceived skill, both of which increase psychological barriers to reentry to organized sport programs or activities (7, 8). Correspondingly, organized sport structures have become increasingly competitive and selective, frequently emphasizing early specialization and elite performance pathways (23), misaligning with broad generational preferences for flexibility and social connection. Kay et al.'s (24) review of youth sport interventions targeting adolescent girls highlighted the limited availability of evidence-based programming designed specifically to sustain girls’ engagement during middle and high school years. Collectively, the body of literature suggests that generational contexts interact with structural sport design to shape participation trajectories. For adolescent girls navigating heightened social evaluation and digital comparison, sport systems that prioritize selection and performance over belonging may amplify their dropout risk.

### Ultimate as a gendered participation context

Ultimate represents a distinctive case within organized sport. The sport emphasizes self-officiation and mixed-gender formats, positioning itself as inclusive and equity-oriented. However, mixed-gender participation structures produced complex gender dynamics. Research on mixed-gender sport indicated that women experienced marginalization, reduced visibility, and pressure to conform to male-dominated styles of play (25). When participation environments failed to ensure physical and psychological safety, women's sustained engagement was compromised.

Emerging and nontraditional sports often lacked robust youth development pipelines, stable school-based infrastructures, and clearly defined reentry pathways. Organizational ecology perspectives suggests that institutional legitimacy and resource stability are critical for sustaining participation over time (26). In the absence of strong institutional anchors, participation becomes particularly vulnerable to disruption—especially during periods of societal instability such as the COVID-19 pandemic. Existing scholarship indicates that women's and girls’ disengagement from sport is rarely attributable to diminished interest (9, 27). Instead, dropout reflected cumulative structural, cultural, and developmental barriers. Applying these insights to Ultimate provided an opportunity to examine how sport organizations could better align participation structures with stated equity values and adapt to post-pandemic and generational realities.

The convergence of post-pandemic disruption, generational change, and persistent gender inequities created a critical juncture for organized sport and for Ultimate specifically. Evidence suggested that women and girls were not disengaging due to lack of motivation, but rather in response to participation environments that failed to sustain belonging, competence, safety, and viable developmental pathways. To examine these dynamics, the following research questions guided this study:

1. How did participation patterns among women and girls in Ultimate change from 2014 to 2024, and how did these trends compare to those of boys and men across youth, collegiate, club, and professional levels?
2. What structural, cultural, and developmental factors influenced women's and girls’ disengagement from and reentry into Ultimate, particularly in the post-COVID participation environment?
3. How did Ultimate's participation structures, visibility practices, and developmental pathways shape women's and girls’ perceptions of belonging, safety, and long-term opportunity within the sport?

## Methodology

### Research design

This study employed a convergent mixed methods design to examine girls’ and women's participation, attrition, and perceptions in Ultimate from 2014 to 2024. A convergent design allows for the simultaneous collection of quantitative and qualitative data, which are analyzed separately and then integrated during interpretation to provide a comprehensive understanding of participation patterns and contextual factors (32). The quantitative survey captured broad trends in participation, social connectedness, barriers, and leadership aspirations, while qualitative interviews provided deeper insight into lived experiences and the mechanisms underlying attrition (see Supplementary Appendix A). To contextualize these findings within the sport's public-facing culture, a social media analysis was also conducted, examining how Ultimate is represented online and whose participation is most visible. Integrating these three approaches enabled the study to identify both the structural and cultural influences on girls’ and women's engagement in Ultimate, highlighting the interplay of social, organizational, and digital factors in shaping retention and inclusion.

Terminology was standardized throughout the manuscript. “Ultimate” refers to the sport, while “ultimate frisbee” is used only when clarifying the sport's common name. “Women,” “girls,” “men,” and “boys” refer to the populations or competition divisions under discussion. “Female,” “male,” and other gender categories refer to respondents’ self-identified survey responses or the categories recorded in the registration data, as specified. “Gender division” refers to a competition category and should not be interpreted as equivalent to self-identified gender. To clarify the relationship among data sources, this study used a multisource mixed-methods design. The registrant data described annual participation patterns across the Ultimate system from 2014 to 2024. The survey examined participants’ reported participation status, social connectedness, perceived barriers, and leadership aspirations. Interviews provided contextual accounts of former participants’ experiences and were conducted with 14 former women participants and one parent during the Fall of 2025. Social-media data described public-facing Ultimate-related content collected from Instagram, X, and Facebook between December 2024 and December 2025. The sources differed in population, timing, sampling frame, and analytic purpose and were therefore not treated as interchangeable estimates of the same population. Findings were integrated during interpretation by comparing areas of convergence, complementarity, and divergence across sources (see Table 1).

**Table 1:** Research design and integration.

Data source	Primary purpose	Sample/Time frame	Analysis	Integration role
USA Ultimate regsitrant data	Describe participation trends	Annual records, 2014–2024	Counts and percentages	Establishes population-level registant patterns
Survey	Examine perceptions and self-reported participation status	1,892 voluntary respondents	Descriptive and inferential analyses	Identifies associations and perceived barriers
Interviews	Explore former participants’ experiences	14 former women participants and one parent	Thematic analysis	Provides contextual explanation and illustrates lived experiences
Social media	Examine public-facing content and visibility	12,460 public mentions; Dec. 2024–Dec. 2025	Sentiment, thematic, gender-associated, and reach analysis	Provides cultural and symbolic context

### Participants

The quantitative survey included 1,892 Ultimate players, of whom 905 identified as female, 924 as male, 50 as non-binary or third gender, and 13 preferred not to disclose their gender. Participants ranged in age from 14 to 68 years (M = 29.01, SD = 12.03) and were predominantly White (74%), followed by Asian (12%), multiracial (6%), and other or undisclosed racial/ethnic identities (8%). Respondents represented a range of competitive levels, including youth, collegiate, club, and professional play, providing a broad perspective on patterns of participation, attrition, and perceptions of inclusivity. Among respondents, 329 participants (176 females, 152 males) reported no longer participating in Ultimate at the time of the survey, enabling analyses of factors associated with dropout and reentry.

Qualitative interviews were conducted with 14 women who had previously participated in Ultimate and subsequently discontinued, along with one parent who provided additional perspective on youth experiences. Participants ranged in age from 18 to 34 years and had experience across multiple levels of play, including high school, collegiate, and club competition. Participants were recruited through purposive and snowball sampling to ensure variation in age, competitive level, and life stage. Semi-structured interviews focused on understanding why participants left the sport, how social and structural factors influenced their engagement, and what challenges or opportunities affected potential reentry.

The social media analysis included publicly available posts from Instagram, X (formerly Twitter), and Facebook over a 12-month period from December 2024 to December 2025. The dataset consisted of 12,460 mentions of Ultimate-related content, with an estimated potential reach of 57.7 million users. Posts were analyzed for sentiment, thematic content, gendered visibility, engagement, and geographic distribution, providing insight into the sport's public-facing culture and the ways in which male and female participation is represented and amplified online.

In this study, administrative registration trends and self-reported current participation were treated as distinct outcomes. Registered participation refers to records in the USA Ultimate registrant dataset. Current participation refers to respondents’ self-reported participation status at the time of the survey. Respondents who reported that they were no longer participating were classified as former or non-current participants for cross-sectional comparisons. As the survey was cross-sectional and did not follow individuals prospectively, these data cannot estimate longitudinal attrition rates or identify temporal predictors of dropout.

### Instrumentation

The survey instrument incorporated validated scales and custom items designed to measure multiple dimensions of participation and engagement. Sense of community was assessed using the McMillan and Chavis (33) scale, which captures participants perceived belonging and connection within Ultimate. Social capital and support were measured using established scales adapted from Côté et al. (23), and motivation and enjoyment were assessed using items derived from self-determination theory (34). Additional survey items addressed perceived inclusivity, experiences of gender-based exclusion, financial and logistical barriers, awareness of Ultimate programming, attitudes toward self-officiating, dropout history, and leadership aspirations. Responses were collected primarily on Likert-type scales, and participants also provided demographic information, competitive level, and current participation status.

Qualitative interviews followed a semi-structured protocol developed to explore participants’ entry into Ultimate, experiences in mixed-gender and women's play, social belonging, injury and safety concerns, structural and logistical barriers, and life transitions influencing participation. Interviews ranged from 45 to 75 min, were audio-recorded with consent, and transcribed verbatim. A parent interview provided additional context regarding youth socialization and programmatic support for girls’ participation.

The social media analysis employed keyword-based data collection to capture posts containing terms such as “Ultimate Frisbee,” “Ultimate,” and “Frisbee.” Metrics included sentiment classification (positive, neutral, negative), thematic coding (e.g., competition, social connection, athleticism), gendered visibility of content, engagement measures (likes, shares, retweets), and geographic location of posts. Automated scraping tools were used in accordance with platform terms of service and ethical guidelines, with personal identifiers removed as appropriate to protect privacy.

### Procedures

Survey participants were recruited through national and regional Ultimate organizations, social media groups, and mailing lists. The survey was administered electronically, and informed consent was obtained prior to participation. Participation was voluntary and responses were anonymized to protect confidentiality. Interview participants were recruited through purposive sampling from survey respondents who reported prior Ultimate experience but were no longer actively participating. Interviews were conducted via secure video conferencing platforms, and participants provided informed consent for audio recording and anonymization of transcripts. The semi-structured protocol allowed for probing and elaboration on emergent topics while maintaining consistency across participants. Social media data was collected from publicly available posts using keyword searches and automated scraping tools. Only posts meeting inclusion criteria related to Ultimate were retained for analysis, and identifying information was removed when necessary. Data collection focused on capturing a full 12-month cycle to account for seasonal variation in competitive play and social media engagement.

### Data analysis

Quantitative survey data were analyzed using SPSS Version 29. Descriptive statistics, including means, standard deviations, and frequencies, characterized participation patterns, social connectedness, barriers, and leadership aspirations. Independent samples t-tests and chi-square tests assessed gender differences in sense of community, social capital, financial constraints, and leadership goals. Temporal trends in attrition were examined by comparing participants’ reported years of disengagement. Effect sizes were reported using Cohen's d for continuous variables and phi coefficients for categorical variables.

Qualitative interviews were analyzed using thematic analysis (35). Transcripts were coded iteratively to identify patterns related to social belonging, mixed-gender dynamics, physical risks, mental health, structural barriers, and life transitions. Codes were grouped into broader themes and triangulated with survey findings to contextualize quantitative results. NVivo 14 software supported coding and analysis, and a second coder reviewed 20% of transcripts to ensure consistency (*κ* = 0.87).

Social media content was analyzed using a mixed quantitative-qualitative approach. Sentiment and engagement metrics were quantified to understand volume and visibility patterns, while thematic coding examined narratives surrounding male and female participation, competition, social connection, and leadership. Gendered visibility patterns were compared with survey and interview results to assess alignment between public-facing representations and women's lived experiences.

### Social media data protocol

Publicly available posts were collected from Instagram, X, and Facebook between December 2024 and December 2025 using *Sprinklr* software operated by the Clemson University Social Media Listening Center (SMLC). Boolean searches used the following terms and hashtags: #Ultimate AND #Frisbee, #UltimateFrisbee, #USAUltimate, #USAU, #WFDF, #LiveUltimate, and #WomeninUltimate. These terms were formulated in collaboration with administrative staff at USA Ultimate—the governing body of Ultimate in the United States—to capture the X posts most saliently concerning Ultimate participation in the United States. The analytic unit was a unique public post. Duplicate posts, reposts, posts unrelated to Ultimate, and non-English/private/other excluded content were removed according to predefined inclusion and exclusion criteria. Given the social-media analysis was conducted by one coder, formal inter-rater reliability was not calculated. To promote consistency, the coder applied a predefined codebook, documented coding decisions, and conducted a duplicate-coding check of 500 posts (4%) after two weeks. Ambiguous posts were coded as *unknown* rather than assigned to a gender or thematic category without sufficient evidence. The social media collection, analysis, and application within this study was consistent with extant scholarly research utilizing such social media data content (e.g., 28–31).

### Integration of methods

A convergent mixed-methods approach enabled the integration of findings from the survey, interviews, and social media analysis to provide a comprehensive understanding of women's participation in Ultimate. Quantitative data established patterns of engagement, attrition, and barriers, while qualitative interviews provided depth by elucidating how social, structural, and life-course factors influenced women's experiences. Social media analysis contextualized these findings within the sport's public-facing culture, revealing disparities in visibility and the prominence of male vs. female participation. Integrating these methods allowed for triangulation of results, increasing the validity and interpretive power of the study, and highlighting the complex interplay of social, structural, and digital influences on girls’ and women's long-term engagement in Ultimate (32).

Integration occurred during interpretation rather than through statistical pooling of the datasets. Registrant data established descriptive participation patterns; survey data summarized reported perceptions and participation status; interviews illustrated how former participants described social, structural, and developmental experiences; and social-media data described public-facing representations of Ultimate. Findings were considered convergent when different sources addressed similar patterns, complementary when one source provided contextual detail about another, and divergent when sources produced different or apparently contradictory findings. For example, higher reported community and social-support scores among women were interpreted alongside women's reports of exclusion and financial barriers rather than as evidence of a single explanatory mechanism.

### Ethical considerations

This research was conducted as part of a funded grant project in collaboration between the researchers and USA Ultimate, the national governing body for Ultimate in the United States. All study procedures were reviewed and approved by the Clemson University Institutional Review Board to ensure adherence to ethical standards. Informed consent was obtained from all participants, and data were anonymized to protect confidentiality. Social media data were collected exclusively from publicly available posts and handled in accordance with established ethical guidelines to prevent identification of individual users.

## Ultimate sport participation patterns: 2014–2024

USA Ultimate provided registrant demographic data spanning 2014 through 2024, enabling an examination of longitudinal participation patterns. A frequency distribution analysis assessed the number and percentage of participants across key demographic and structural variables over time. Variables were selected for their relevance to participation pathways and included gender, race, membership state, competition level, gender division, team competition level, and event type. Although the dataset contained additional variables, these categories provided the clearest insight into structural participation dynamics within the Ultimate ecosystem. Data were analyzed descriptively to identify stable patterns, emerging trends, and notable shifts across the eleven-year period.

### Gender representation in ultimate participation

Across the full period of analysis, Ultimate participation exhibited a persistent and substantial gender imbalance. From 2014 to 2024, male participants consistently outnumbered female participants by roughly two to one. On average, 65.7% of participants identified as male, 33.2% identified as female, and a comparatively small proportion identified as nonbinary, preferred not to report, or were categorized as UN; collectively, these latter categories accounted for a minimal share of total participation (see Figure 1). The gender distribution remained remarkably stable across the eleven-year period, suggesting that disparities were not attributable to short-term fluctuations but reflected enduring structural patterns. Despite broader discourse on gender inclusion in sport and policy-level advances in women's athletic opportunities (17, 18), the overall gender composition of Ultimate participants changed little over time. These findings align with broader research documenting persistent gender gaps in organized sport participation, particularly during adolescence (6).

**Figure 1:**
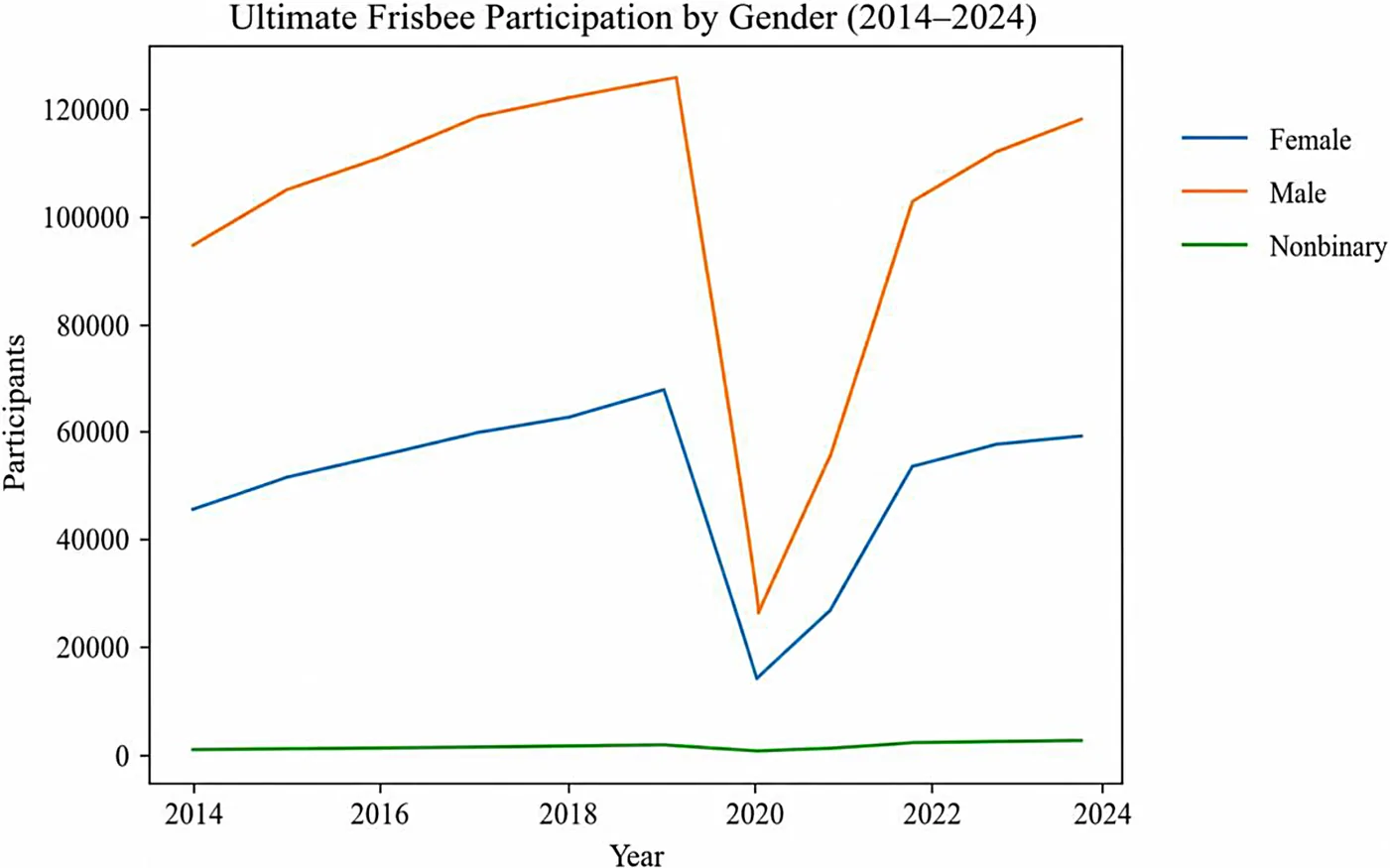
Ultimate participation by gender (2014−2024).

### Racial composition of ultimate participants

Analysis of race-based participation data indicated that Ultimate participants were predominantly White, with limited diversification across the study period. On average, White participants accounted for 64.3% of total participation. The proportion of White participants increased over time, rising from 47.2% in 2014 to approximately 70% in peak years such as 2019 and 2022 (see Figure 2). Asian participants represented the second-largest racial group, averaging 9.1% of total participation. Participation in this group demonstrated a steady upward trend, increasing from 5.4% in 2014 to 11.6% in 2024. Participation among other racial categories increased modestly but remained comparatively small and did not meaningfully alter the overall racial composition. These trends suggest that growth in Ultimate participation occurred primarily within already-represented populations rather than through broad-based diversification. This pattern is consistent with research indicating that access to emerging and nontraditional sports often reflects existing social networks, institutional exposure, and resource distribution (26). The limited diversification observed over the decade highlights questions regarding differential access, visibility, and entry points across racial and ethnic communities.

**Figure 2:**
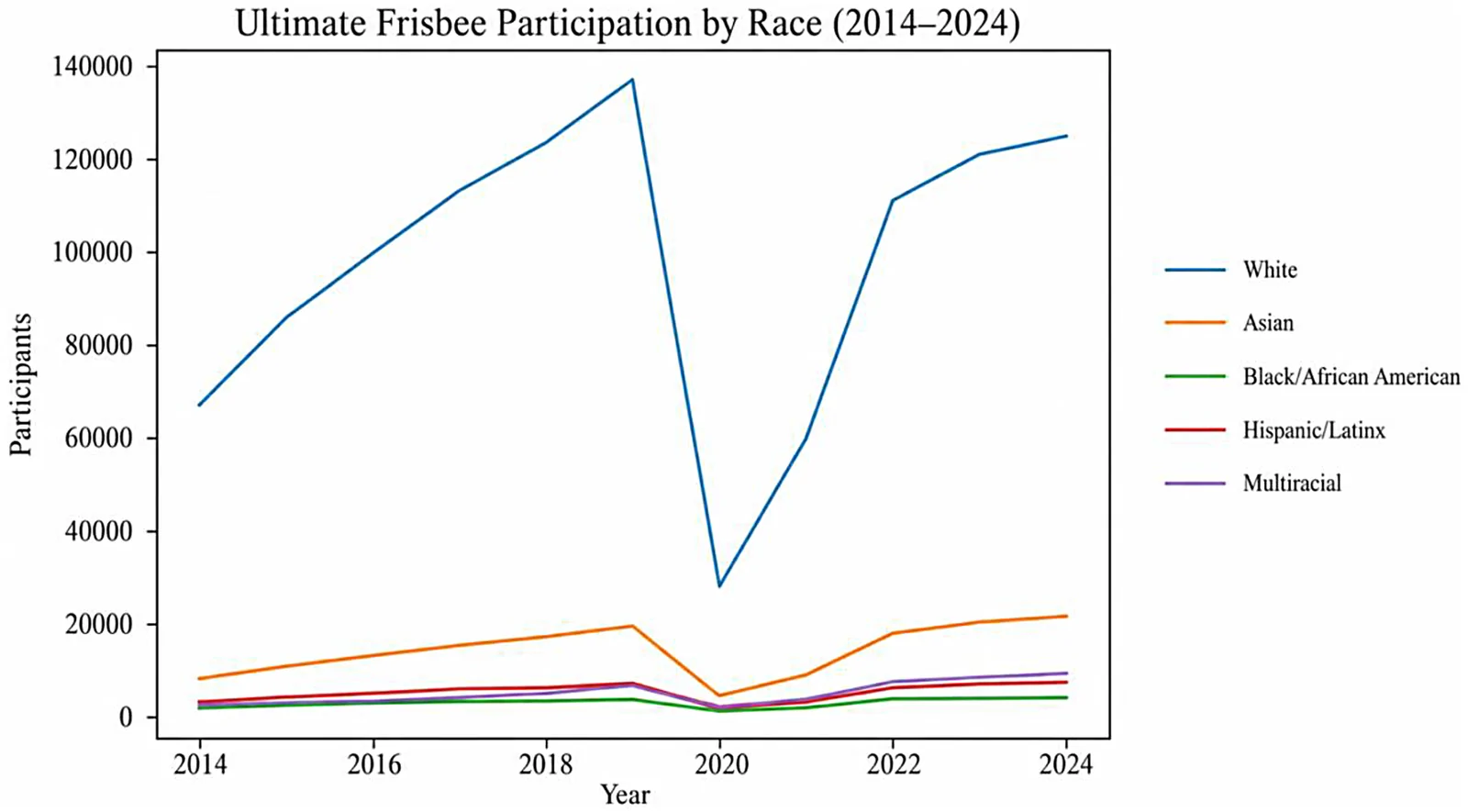
Ultimate participation by race (2014−2024).

### Geographic concentration of participation

Participation in Ultimate was highly geographically concentrated. Across all years, a small number of states consistently accounted for a disproportionate share of participants, while the majority of states contributed less than 1% of annual participation (see Figure 3). Depending on the year, between 34 and 40 states fell below the 1% threshold, indicating substantial regional disparities. California and New York ranked among the top five membership states in every year from 2014 to 2024. Massachusetts and North Carolina appeared in the top five in all but one year, underscoring the stability of established participation hubs. Minnesota ranked among the top five states in seven of the eleven years analyzed, and Colorado emerged as a top five state for five consecutive years (2020–2024). Georgia appeared in the top five in only one year.

**Figure 3:**
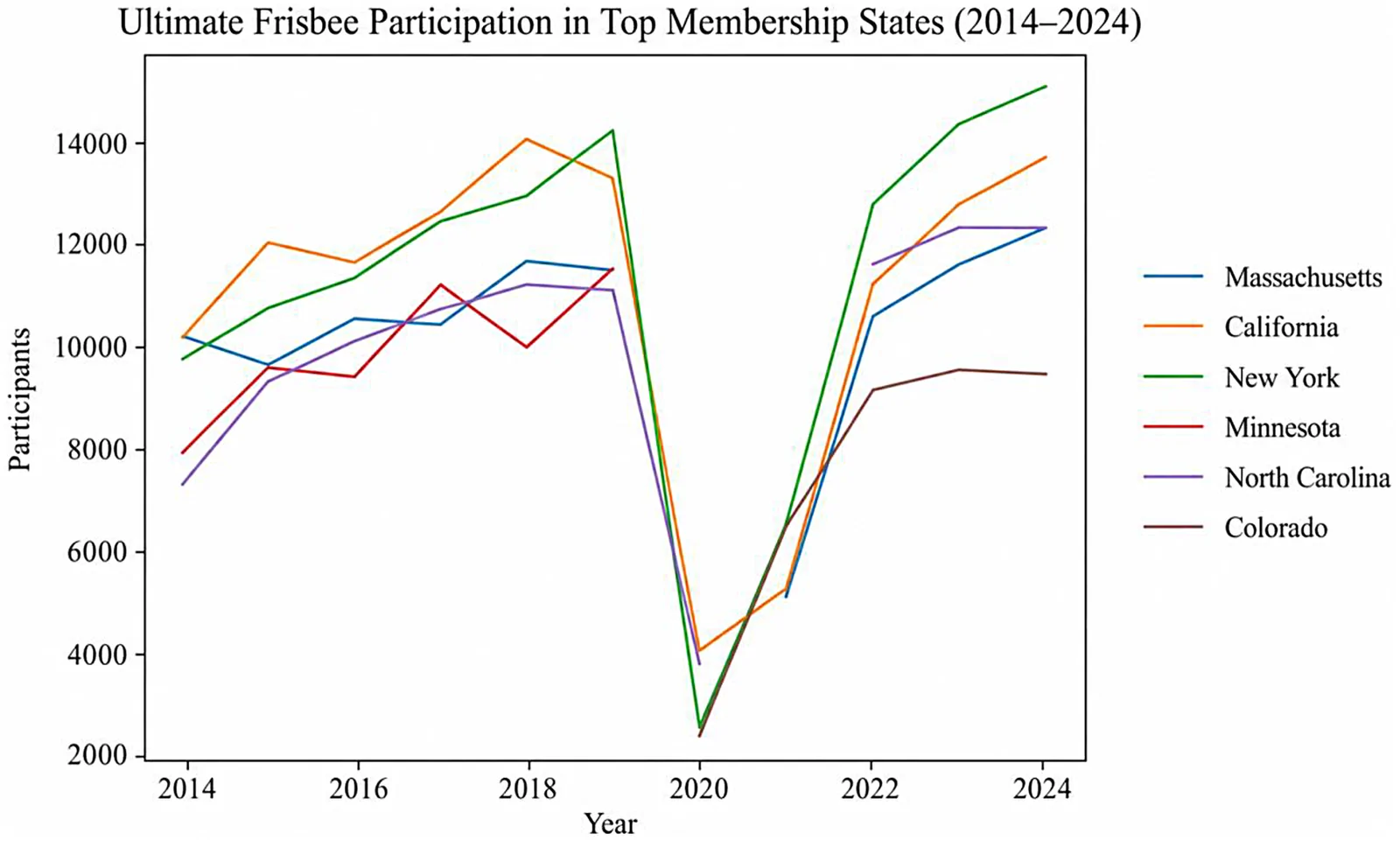
Ultimate participation in Top membership states (2014−2024).

These patterns indicate that Ultimate participation remained anchored in a limited number of established regions characterized by strong infrastructure, institutional support, and a historical presence of the sport. Organizational ecology perspectives suggest that institutional stability and concentrated resource environments facilitate sustained participation (26). Conversely, sparse participation across much of the country may have constrained youth exposure and limited opportunities for growth outside established hubs.

### Participation across competition levels

Participation patterns across competition levels revealed both structural expansion and concentration. In 2014, participants were distributed across ten competition levels; by 2024, this number had expanded to twenty, reflecting increased structural differentiation within the sport. However, not all competition levels were available annually, and participation remained heavily concentrated within a small subset of levels (see Figure 4). Club, college, and high school levels consistently accounted for the majority of participants, averaging 35.7%, 36.3%, and 16.4% of total participation, respectively. All other levels attracted substantially smaller shares, with several accounting for less than 1% of total participation.

**Figure 4:**
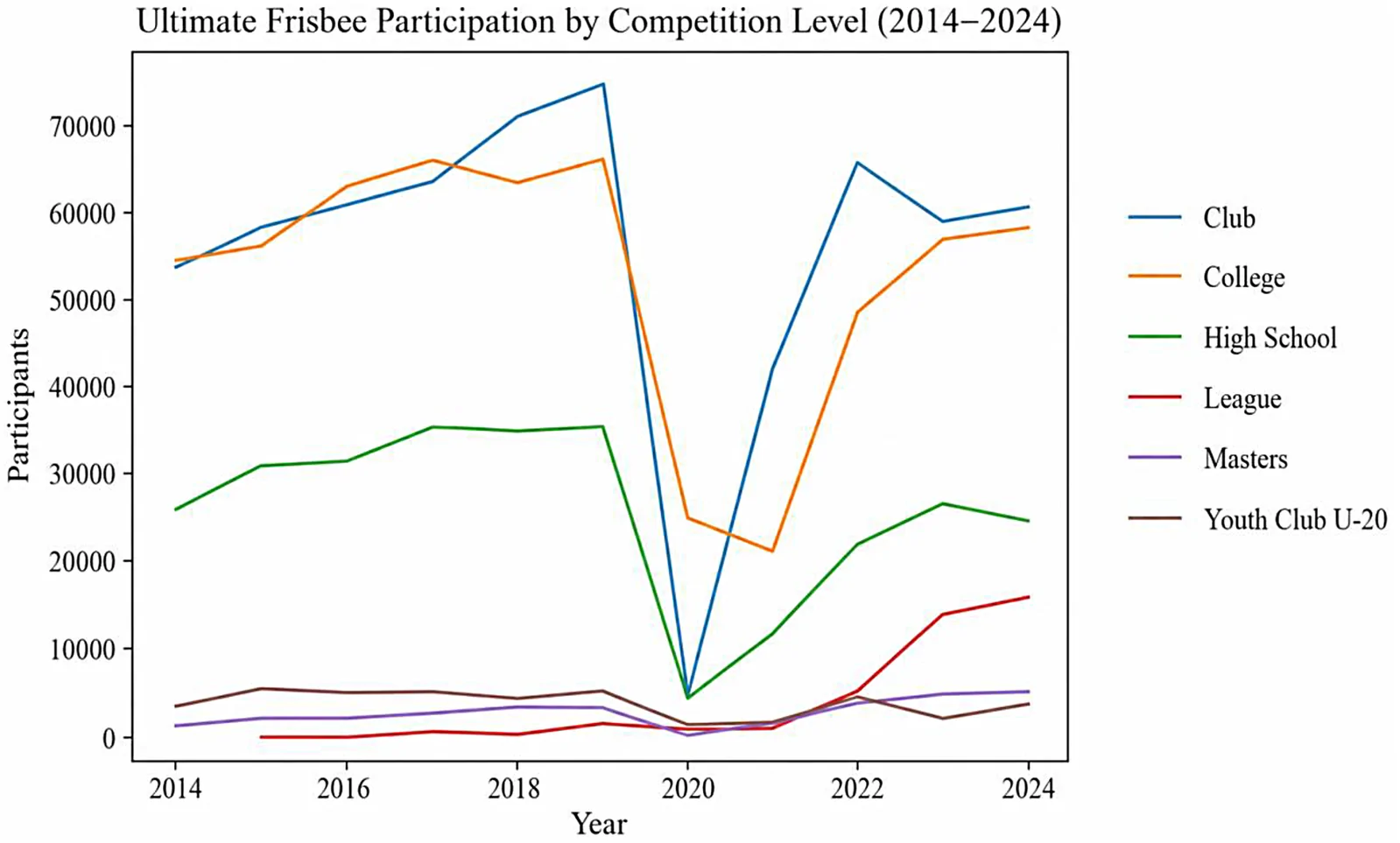
Ultimate participation by competition level (2014−2024).

One notable exception was the league competition level, which accounted for less than 1% of participants in 2015 but increased steadily over time, reaching 8.8% of total participation by 2024. Such growth suggests rising demand for competition formats offering flexibility, reduced time commitments, or lower barriers to entry compared with traditional club and tournament-based structures. These trends mirror broader youth sport research highlighting post-pandemic shifts toward less intensive and more socially oriented participation formats (1, 2).

### Gender division participation patterns

Participation by gender division revealed pronounced and persistent disparities, particularly in youth divisions (see Figure 5). Across all years, boys’ division participation exceeded girls’ division participation by a factor of three to four, averaging 11.7% of total participants for boys and 3.4% for girls. Both divisions experienced declines over time, with boys’ participation decreasing from 13.9% in 2014 to 10.3% in 2024, and girls’ participation falling from approximately 4.3% in 2015–2016 to 2.7% by 2024. These parallel declines indicate contraction in the youth participation base overall, with girls consistently underrepresented. Gender imbalances persisted in adult divisions as well. Men's division participation averaged 35.6%, nearly double the women's division average of 18.7%. In contrast, the mixed division represented a substantial and growing share, averaging 30.2% overall and increasing steadily from 25.8% in 2014 to 32.2% in 2024. By the latter years of the dataset, men's and mixed divisions together accounted for the majority of participation. The expansion of mixed formats reflects structural shifts within the sport and raises questions regarding how mixed participation environments intersect with women's long-term retention, an issue explored in subsequent analyses.

**Figure 5:**
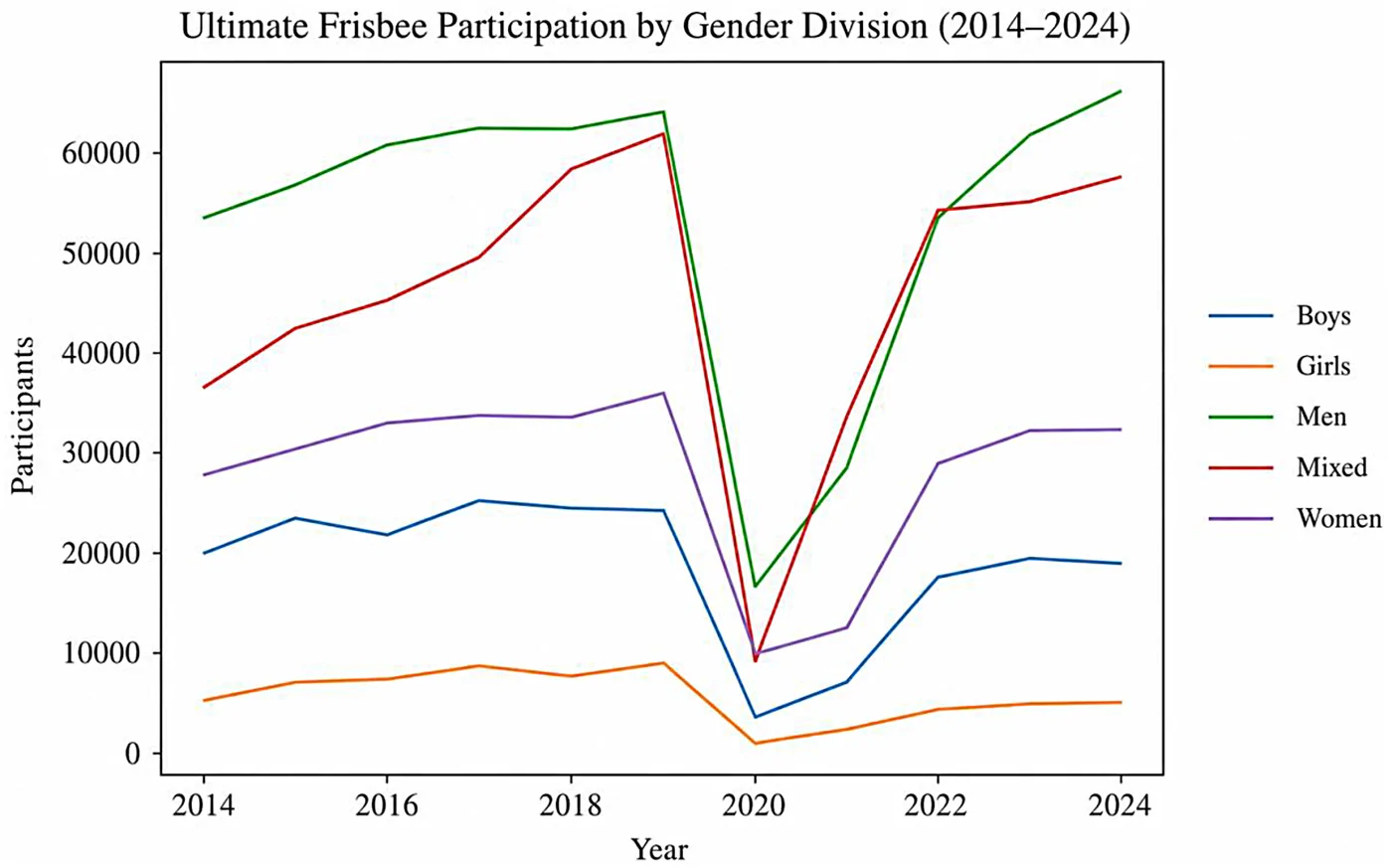
Ultimate participation by gender division (2014−2024).

### Team competition levels & youth participation trends

Examining team competition levels provided a more granular view of gender disparities (see Figure 6). Participation rates for boys and men were approximately two to three times higher than those for girls and women at both high school and club levels. At the college level, men's participation remained nearly double that of women. High school participation trends were particularly notable. Boys’ participation declined from a peak of 12.2% in 2015 to 7.3% in 2024, while girls’ participation followed a similar but consistently lower trajectory, decreasing from nearly 4% between 2015 and 2019 to approximately 2% from 2021 through 2024. These sustained declines suggest contraction in the youth pipeline beyond short-term pandemic effects and align with broader evidence of adolescent sport dropout during this developmental period (14). Simultaneously, newer team competition formats demonstrated emerging growth. League mixed teams, introduced more recently, increased participation over the past four years, suggesting shifting preferences toward formats perceived as more accessible or socially oriented. Other competition levels exhibited year-to-year variation but did not demonstrate consistent directional trends.

**Figure 6:**
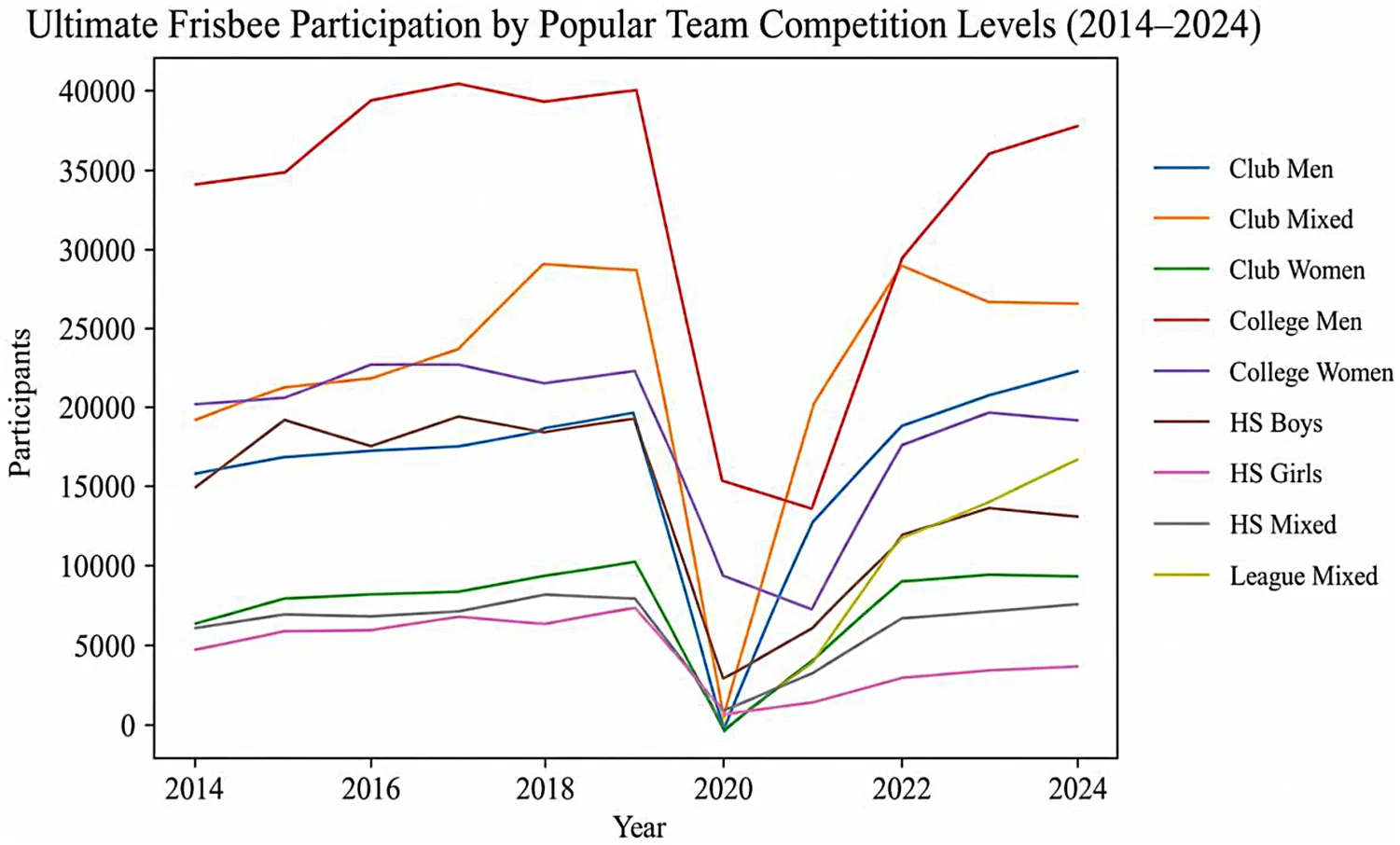
Ultimate participation by popular team competition levels (2014−2024).

### Participation by event type

Participation varied meaningfully by event type (see Figure 7). Regional championship tournaments, sanctioned club regular season tournaments, and sanctioned college regular season tournaments consistently attracted the largest shares of participants, averaging 11.1%, 12.9%, and 18.2% of total participation, respectively. In contrast, insured practices, tryouts, and scrimmages (4.0%), national championship tournaments (3.9%), and state championship tournaments (4.9%) accounted for smaller proportions. Several event types exhibited notable shifts over time. Conference championship tournament participation declined modestly, from 9.7% in 2014 to 8.0% in 2023–2024, while state championship participation declined more substantially, falling from 7.0% to 4.0%. Conversely, participation in insured practices, tryouts, and scrimmages increased from roughly 2% in 2014–2015 to 5.4% in 2024, and national championship participation increased slightly. These shifts suggest recalibration of engagement toward developmental or preparatory events relative to traditional championship pathways.

**Figure 7:**
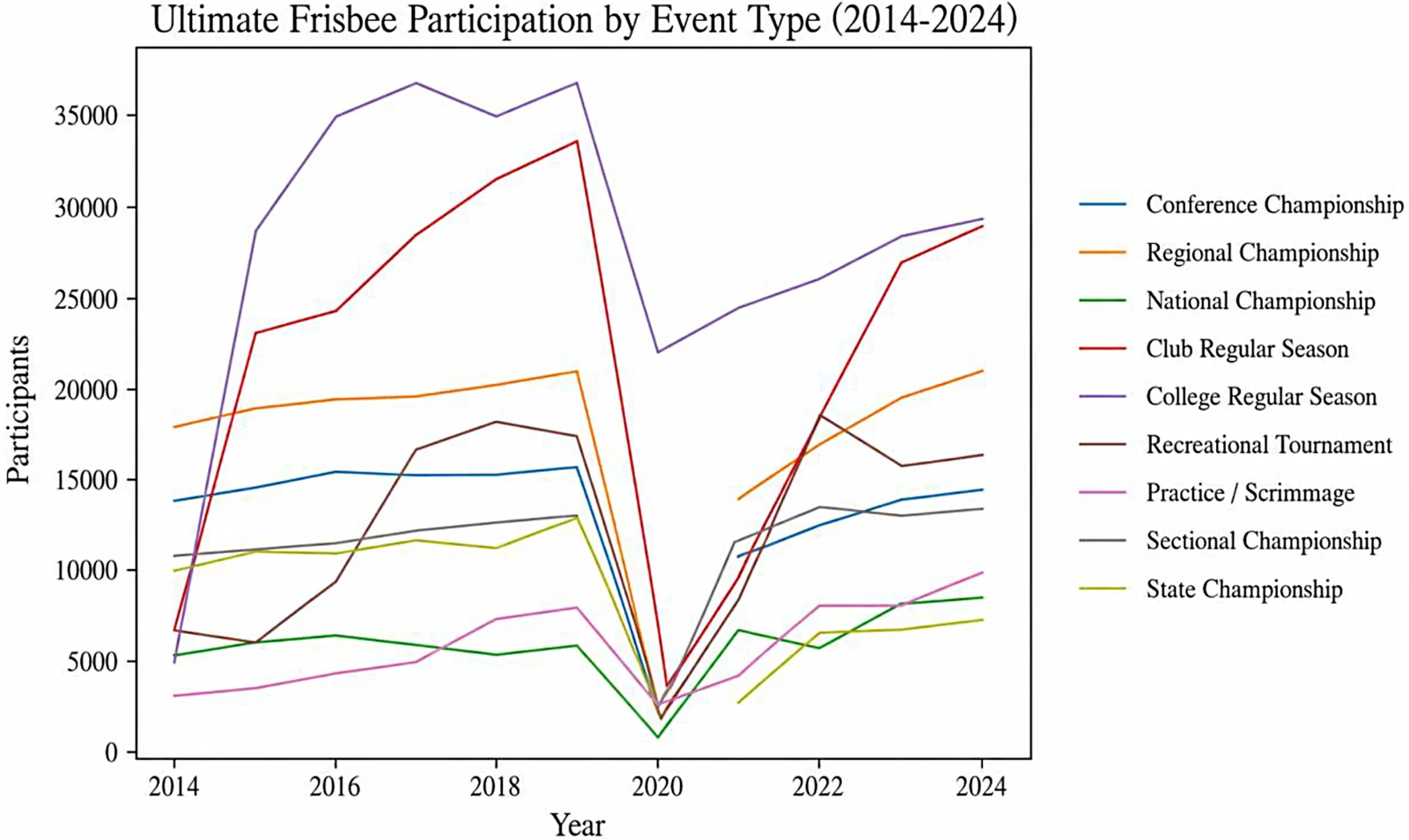
Ultimate participation by event type (2014−2024).

Collectively, the findings reveal several consistent patterns in Ultimate participation from 2014 to 2024 (i.e., persistent gender imbalance, limited racial diversification, geographic concentration within established hubs, declining youth participation, growing interest in league-based and mixed-gender formats). Although the sport expanded structurally through additional competition levels and event types, this expansion did not correspond with strengthened gender equity or a more robust youth pipeline. These descriptive patterns provide critical context for interpreting women's and girls’ disengagement from Ultimate. The persistence of gender disparities, contraction of youth divisions, and growth of mixed and lower-commitment formats suggest that broader post-pandemic youth sport dynamics may have interacted with existing structural characteristics of Ultimate. The following section builds on these patterns by examining the lived experiences and perceptions underlying these participation trends.

## Quantitative survey results

### Participation, belonging, & barriers

An electronic survey of 1,892 Ultimate players captured a diverse sample, including 905 females, 924 males, 50 non-binary or third-gender individuals, and 13 respondents who preferred not to disclose their gender. The majority identified as White (74%), followed by Asian (12%) and multiracial (6%), with a mean age of 29.01 years (SD = 12.03). Respondents reported playing Ultimate an average of 2.7 days per week (SD = 1.27). Among the sample, 329 participants (176 females and 152 males) indicated that they were no longer actively participating. Female participants reported a significantly higher sense of community (M = 37.51, SD = 5.90) than males (M = 36.90, SD = 6.36), t(1892) = −2.04, *p* = .041, and higher social capital and support (M = 30.33, SD = 4.43 vs. M = 29.62, SD = 4.84), t(1892) = −3.15, *p* = .002. No significant gender differences emerged for motivation and enjoyment (females M = 26.37, SD = 4.00; males M = 26.59, SD = 4.04; t(1892) = 1.14, *p* = .254), indicating that women's engagement is perhaps driven more by social connection than intrinsic enjoyment (36, 37).

Despite widespread agreement that Ultimate is “an inclusive community” (females 85.7%; males 84.4%), 53.8% of women reported feeling “excluded or overlooked” during play, compared with 32% of men, *χ*^2^(1, *N* = 1829) = 104.36, *p* < .001. Financial barriers were also more salient for women: 49.7% reported they would participate more if costs were reduced, compared to 30.9% of men, *χ*^2^(1, *N* = 1829) = 42.56, *p* < .001. Leadership aspirations further highlighted gender differences, with 70% of males versus 58.7% of females expressing interest in coaching or other leadership roles. Although recent annual attrition rates showed increases among females in 2024–2025, most participants planned to continue playing over the next year (males 84.2%; females 80.9%). These results suggest that women's attrition from Ultimate is not attributable to lower motivation or enjoyment. Instead, social, structural, and financial factors—particularly subtle exclusion, limited leadership opportunities, and resource constraints—appear to shape sustained engagement and dropout (38, 39).

## Qualitative perspectives

To provide contextual depth to the quantitative findings, in-depth interviews were conducted with fourteen women who previously participated in Ultimate and subsequently discontinued, along with one parent. These interviews illuminated the complex social, structural, and physical factors influencing attrition.

### Entry pathways & early experiences

Participants typically began playing Ultimate in adolescence or early adulthood, often through school-based teams, physical education classes, or peer networks. Very few described early childhood participation, emphasizing the sport as a late-entry activity. Early experiences were formative; several participants recounted negative encounters with mixed-gender dynamics that disrupted participation: “Boys never threw to girls” (Participant 13). These experiences align with survey reports of women feeling overlooked despite broader perceptions of inclusivity.

### Social belonging & community

Social connections emerged as a central determinant of participation. One participant explained, “Once those upperclassmen girls graduated, it got harder. They were the reason I stayed” (Participant 3), while another reflected, “I think I stayed because of the people more than anything else” (Participant 11). These narratives provide qualitative depth to the quantitative finding that women report higher sense of community and social support. Disruptions to these social networks—through graduation, relocation, or team dissolution—were consistently linked to attrition.

### Pandemic-related disruption

The COVID-19 pandemic served as a pivotal interruption. Several participants described Ultimate as a critical social outlet during lockdown: “During COVID, that was basically my only social outlet… probably the peak of me being involved in Frisbee” (Participant 6). Post-pandemic, some participants encountered diminished infrastructure and social momentum, making reentry challenging: “The team didn't exist anymore… and then the inertia was gone” (Participant 12). These experiences mirror broader trends in pandemic-related sport disengagement (46).

### Mixed-gender participation & safety

Mixed-gender play posed ongoing challenges to confidence and safety. Participants described feeling physically at risk and socially marginalized: “A lot of times when playing mixed, you’re kind of just playing men's ultimate” (Participant 13), and “I never had a chance to be a good matchup. It just felt unfair” (Participant 4). These qualitative accounts reinforce survey findings of women experiencing subtle exclusion despite the perception of an inclusive community.

### Physical injury & mental health

Injury emerged as a critical factor in attrition, especially when combined with fear of re-injury and inadequate support. One participant recounted, “A guy ran straight into me, and I tore my ACL completely. It was really traumatic” (Participant 4). Emotional exhaustion and competitive pressure were also common: “It wasn't fun anymore if I felt bad going to practice” (Participant 5). These findings align with prior research linking injury and burnout to dropout (40, 41).

### Structural barriers & reentry challenges

Post-college structural barriers, including limited women-only options, inconsistent commitment expectations, and unclear entry points, hindered sustainable participation. One participant explained, “Club just didn't feel as legitimate…no coach, variable commitment” (Participant 1). Another described the lack of appropriate competition levels: “Pickup was either way too intense or not serious at all” (Participant 10). Financial considerations compounded these challenges, consistent with survey data indicating cost as a limiting factor. The parent interview reinforced these themes, noting the importance of belonging over skill development and the absence of adult-led inclusive norms: “No coaches or program leaders intervened…she simply reallocated her time to other activities.” Qualitative themes complemented survey data by explaining why women, despite higher motivation and enjoyment, report attrition. Social disruption, mixed-gender challenges, injury, mental health strains, and structural barriers all interact to shape women's participation trajectories and reentry opportunities.

## Social media analysis

To examine structural and cultural context, a social media analysis of Instagram, X (formerly Twitter), and Facebook was conducted over a 12-month period (December 2024–December 2025), analyzing 12,460 mentions with an estimated reach of 57.7 million users.

### Sentiment & content themes

Sentiment toward Ultimate was overwhelmingly positive, with 97.1% of mentions classified as favorable. Content emphasized competition, athleticism, team identity, tournaments, and enjoyment, using terms such as “ultimate,” “frisbee,” “team,” and “fun.” Video posts accounted for 42% and photo-based posts for 30% of engagement, primarily highlighting game moments, tournament plays, and team celebrations (42). Despite overall positivity, gender disparities were pronounced. Male-associated content generated 635 mentions versus 274 for female-associated content, with top influencers largely organizational, professional, or male-focused. Women-led initiatives and hashtags represented a small fraction of total engagement, echoing survey findings of reduced leadership aspirations and qualitative reports of subtle exclusion (17, 38). Engagement was primarily United States based, suggesting that dominant narratives about competitiveness, pathways, and visibility reflect United States centric norms and may obscure international perspectives (43).

The social media findings provide structural context for both survey and qualitative insights. While the sport is portrayed as enjoyable and socially rewarding, men's participation and elite-level competition dominate visibility, potentially shaping perceptions of belonging, legitimacy, and long-term opportunity for women and girls (44, 45).

## Discussion

The present study integrated quantitative registrant data, qualitative interviews, and social media analysis to examine women's and girls’ participation in Ultimate from 2014 to 2024. Given the study was observational and included a cross-sectional voluntary survey, a small purposive interview sample, descriptive registrant analyses, and public social-media data, the findings should not be interpreted as establishing causal explanations for participation decline or dropout. Registrant data describes changes in participation counts and proportions; survey data identify associations among reported perceptions and participation status; interviews represent participants’ accounts; and social-media data describe publicly visible content. Across sources, the findings are consistent with the possibility that social, structural, financial, cultural, and developmental conditions are related to participation, but they do not demonstrate that these factors caused disengagement or explain population-level trends.

Findings indicate that attrition among female participants analyzed in this study in not driven by a lack of interest or enjoyment but rather by the interplay of structural, cultural, and developmental factors. Survey data revealed that social support and community may be central to retention, while interviews highlighted the influence of mixed-gender dynamics, injury, life transitions, and limited reentry pathways on disengagement. Social media analysis provided additional context, showing that public-facing discourse often amplifies men's participation and leadership, creating symbolic and visibility-related inequities. Collectively, these results suggest that sustainable participation among women and girls requires comprehensive strategies addressing inclusivity, social connection, flexible pathways, financial accessibility, and leadership development.

Longitudinal participation patterns reveal persistent gender disparities, limited racial diversification, geographic concentration in established hubs, and declining youth engagement, particularly within high school divisions. These trends occurred despite structural expansion in competition levels and growth of mixed-gender and league-based formats, indicating that providing access alone is insufficient to promote equitable participation. These patterns align with prior research on adolescent sport participation, which consistently identifies social evaluation, perceived competence, and structural barriers as primary drivers of girls’ sport dropout, rather than lack of motivation (6, 9, 10).

Qualitative findings underscore that girls often perceive competitive and mixed-gender environments as psychologically risky. One participant reflected, “I enjoy playing, but sometimes it feels like I have to match the boys’ style of play, and it's exhausting,” highlighting the tension between inclusion in mixed formats and sustainable engagement. These insights extend previous literature by demonstrating that even equity-oriented sports can reproduce gendered participation patterns when structural and cultural mechanisms do not adequately support developmental and social needs.

The study also advances understanding of structural inequities in youth sport. Racial composition data indicate that growth occurred primarily within already-represented populations, with White participants constituting the majority and Asian participation increasing modestly over time. This aligns with research showing that access to emerging and nontraditional sports is influenced by social networks, institutional exposure, and resource availability (26). Geographic concentration within a limited number of states further illustrates the role of infrastructure and organizational ecology in sustaining participation, emphasizing the uneven distribution of structural supports across regions. However, geographic concentration simultaneously constrains interpretation of the findings. As a large share of registrants come from established Ultimate hubs, the observed participation patterns may reflect the infrastructure, institutional partnerships, coaching capacity, transportation options, and competition opportunities available in those regions. Areas with less established infrastructure may have lower registrant counts because opportunities to learn about, access, and sustain participation in Ultimate are less available or less visible rather than merely a lack of interest. Correspondingly, these findings may describe established participation ecologies more strongly than conditions in less-developed regions, and generalization beyond these hubs should be made cautiously.

Mixed-gender and low-commitment league formats emerged as promising areas of growth. The expansion of these formats may reflect generational preferences for flexibility, social connection, and reduced competitive pressure among Generation Z and Generation Alpha athletes (20, 24). However, while these formats can enhance engagement, they do not automatically reduce gender disparities, as women and girls remain underrepresented in high school and club divisions. These findings underscore the necessity of intentional structural interventions that prioritize equity, developmental support, and psychological safety alongside opportunities for belonging.

Social media analysis provides further insight into the cultural and symbolic dimensions of participation. Women frequently referenced a lack of role models and leadership representation as discouraging, reflecting broader literature indicating that limited visibility of female athletes and coaches constrains long-term engagement and identification opportunities (17, 18). These findings reinforce the importance of multi-dimensional strategies that simultaneously address structural, cultural, and symbolic factors to support retention and equitable participation.

## Practical recommendations

The findings suggest several possible practice implications for sport managers, organizational leaders, and youth sport practitioners. Given this study did not evaluate interventions, the recommendations below should be understood as informed hypotheses for program development and future testing rather than as evidence-based prescriptions. Women and girls-only programming, coaching education, rule adaptation, mentorship, regional development, and visibility strategies may warrant evaluation as approaches to supporting belonging, safety, skill development, and continued participation.

Mixed-gender formats require careful attention to ensure equitable experiences. Coaching education, rules adaptation, and explicit attention to role visibility and scoring opportunities can mitigate gendered pressures, allowing women and girls to participate safely and competitively without conforming to male-dominated norms. Geographic disparities in participation highlight the need for regional development, partnerships with schools, and outreach to underrepresented communities to expand access and promote diversity (4, 26). Visibility of female athletes, coaches, and leaders should also be prioritized in both digital and community spaces. Promoting narratives of female participation, highlighting equitable competitive achievements, and showcasing leadership pathways can reinforce cultural inclusion, support identification, and challenge symbolic barriers (17, 18). Finally, ongoing evaluation of program efficacy is essential. Collecting quantitative and qualitative data on participation, engagement, and perceptions can guide real-time adjustments to interventions and ensure that programs effectively meet the needs of targeted populations.

## Limitations

Several limitations should be considered when interpreting these findings. The voluntary survey may overrepresent engaged participants and is cross-sectional, preventing estimation of longitudinal attrition or temporal predictors of dropout. The survey, registration, interview, and social-media sources also differed in sampling frame, timing, and measurement, and possible overlap existed because some interview participants were recruited from survey respondents. The small purposive qualitative sample, including only one parent, provides contextual accounts rather than generalizable estimates of former participants’ or parents’ perspectives. Registration data may not capture informal, unregistered, or recreational participation, and changes in registration categories or data-collection procedures over time may affect comparisons. Social-media findings are limited by uncertainty concerning coding procedures, gender classification, sentiment classification, and estimated-reach metrics. Unmeasured confounding by age, competitive level, current participation, race/ethnicity, geography, and years of experience may affect survey comparisons. These observational data cannot establish causality. Finally, the concentration of participation in established regions and the focus on USA Ultimate and the United States limit generalizability to less-developed regions, other sports, and other national contexts.

In addition, generational labels are used throughout this manuscript only as contextual background for the period examined. The present study did not measure generational identity, cohort effects, screen time, mental-health status, or generational preferences. Age, period, and cohort effects also cannot be disentangled using the available data. Therefore, generational characteristics are not treated as explanations for the observed participation patterns. Future research should directly measure age, cohort, technology use, and participant preferences before drawing conclusions about generational influences on sport engagement. Despite these constraints, the mixed-methods design facilitated a nuanced understanding of patterns, perceptions, and structural mechanisms influencing women's and girls’ participation.

## Future research

Further research is needed to examine the effectiveness of interventions aimed at increasing participation among women and girls in emerging sports. Longitudinal studies could assess the impact of structural reforms, mixed-gender programming, mentorship initiatives, and youth development pipelines on retention, skill acquisition, and psychological safety over time. Comparative studies across sports, regions, and countries would clarify how institutional support, cultural norms, and socioeconomic factors shape participation trajectories. Investigating the experiences of underrepresented racial and ethnic groups, as well as nonbinary and gender-diverse athletes, could provide critical insights into inclusion and equity. Utilizing direct observation, digital ethnography, or participatory research methods may enhance understanding of how social and relational dynamics interact with structural features to influence engagement. Research that explores the intersection of generational preferences, technology use, and sport design could inform developmentally appropriate approaches to sustain youth participation in evolving organized sport contexts.

## Conclusion

This study offers comprehensive insight into women's and girls’ participation in Ultimate from 2014 to 2024, highlighting persistent gender disparities, limited racial diversification, geographic concentration, and declining youth engagement. Integrating quantitative registrant data with qualitative interviews and social media analysis revealed that structural, cultural, and developmental factors collectively influence engagement and disengagement. The expansion of mixed-gender and low-commitment league formats offers promising avenues for increasing accessibility; however, these innovations alone do not eliminate underlying inequities. Aligning participation pathways with developmental needs, fostering inclusive cultures, and expanding access to underserved regions are essential to sustaining equitable participation. By situating Ultimate within broader post-pandemic and generational trends, this research advances understanding of how emerging sports can foster inclusive, sustainable, and engaging pathways for women and girls while contributing to scholarship on youth sport, gender equity, and organizational sustainability.

## Data Availability

The raw data supporting the conclusions of this article will be made available by the authors, without undue reservation.
