# Supplementary material for: Post-pandemic trends in youth sport: structural and social determinants of girls’ and women's engagement in Ultimate
Source: Front Sports Act Living. 2026 Sep 15;8:1958579. doi: 10.3389/fspor.2026.1958579 (PMC13621784; doi:10.3389/fspor.2026.1958579)
Supplement: Supplementary file 1 [file Supplementaryfile1.docx]

**Supplementary Appendix**

*Demographics & Participation Background*

1. What is your gender identity? [male, female, other, prefer not to say]
2. What is your current age?
3. Are you currently an active participant in Ultimate?
   1. [IF NOT] In what year did you stop participating?
4. What is your race?
5. In what state(s) do/did you participate in Ultimate?
6. How many miles do you travel to participate in Ultimate?
7. At what age were you exposed to Ultimate? [SLIDER SCALE]
8. For how many years have you played Ultimate? [SLIDER SCALE]
9. Are you currently a member of a club/recreational Ultimate team? [Y/N]
   1. [IF YES] Which best describes the competitive level you currently play? [recreational, competitive amateur league, professional league, college/university, other, not sure]
   2. How did you first learn about your current Ultimate team? [OPEN RESPONSE]
   3. On a scale of 1-5, how welcoming was the team when you first joined? [SLIDER SCALE – 1=not at all; 5-very welcoming]
10. How many days per week do you play Ultimate? [1-7 SLIDER SCALE]
11. Do you follow USA Ultimate accounts on social media? [Y/N]
12. How often do you search for news or competitive results from USA Ultimate sanctioned events? [SLIDER SCALE – 1=not at all; 5=very often]
13. Do you play any sports other than Ultimate? [OPEN RESPONSE]

*Sense of Community in Ultimate (modified from Warner et al. (2013) Sense of Community in Sport (SCS) Scale).*

*5-point Likert Scale (1=strongly disagree, 2=disagree, 3=neither agree nor disagree, 4=agree, 5=strongly agree)

When considering your participation in Ultimate:

- I feel like I belong when playing Ultimate.
- I can rely on other Ultimate players to support me when needed.
- I feel my opinions are valued among Ultimate players.
- Ultimate players treat everyone fairly regardless of skill level.
- I often socialize with other Ultimate players outside of practice or games.
- Ultimate players share common interests and goals.
- I feel comfortable and safe when playing Ultimate.
- The competitive atmosphere within Ultimate motivates me to improve.
- I would describe Ultimate as “my community.”

*Social Capital & Support in Ultimate (modified from Paldi et al. (2021) study on social capital as a mediator in team sport environments).*

*5-point Likert Scale (1=strongly disagree, 2=disagree, 3=neither agree nor disagree, 4=agree, 5=strongly agree)

When considering your participation in Ultimate:

- I have made new friends through participating in Ultimate.
- Because of Ultimate, I am more connected to people in my community.
- I trust Ultimate players to treat me with respect.
- I feel I can ask other Ultimate players for help or advice.
- I participate in social activities with Ultimate players (e.g., meals, socializing).
- Ultimate players look out for one another.
- Ultimate helps me feel like I matter and am contributing to something bigger than myself.
- I feel judged or misunderstood by others when I tell them I play Ultimate.
- I worry about how I will be perceived socially as an Ultimate player.

*Motivation & Enjoyment of Ultimate (modified from McDonough & Crocker (2005) study on participant motivation in sport).*

*5-point Likert Scale (1=strongly disagree, 2=disagree, 3=neither agree nor disagree, 4=agree, 5=strongly agree)

When considering your participation in Ultimate:

- I play Ultimate because I genuinely enjoy it.
- I feel excitement to go play Ultimate.
- Even without a formal competitive structure, I would continue playing Ultimate.
- Playing Ultimate makes me feel physical competent and confident.
- The friendships I have built playing Ultimate keep me engaged in the sport.
- If I had to quit playing Ultimate, I would miss it tremendously.

*Barriers, Inclusion, and Feelings towards Ultimate Participation (modified from Eagleman (2013), Eime et al. (2013), and Hopkins et al. (2022) studies on factors associated with sport participation).*

*5-point Likert Scale (1=strongly disagree, 2=disagree, 3=neither agree nor disagree, 4=agree, 5=strongly agree)

When considering your participation in Ultimate:

*INCLUSIVITY BARRIERS*

- My peers encourage me to participate in Ultimate.
- I feel Ultimate is an inclusive community.
- There are times when I have felt excluded or overlooked when playing Ultimate.
- My family and/or friends support my participation in Ultimate.
- I face fewer barriers to participate in Ultimate than others in my community.

*GENDER BARRIERS (If indicated they were a girl/woman in demography section)*

- I sometimes feel unsure if I *fit in* with Ultimate players because of my gender.
- I feel Ultimate is inclusive of girls and women, specifically.
- I am confident that Ultimate provides equal opportunities for girls and women to participate and lead within the sport.
- Some people in my social circle think Ultimate is “not for girls.”
- There are stereotypes about girls and women who play competitive or contact sports like Ultimate.
- I have been discouraged from playing Ultimate because of my gender.
- I feel empowered to challenge traditional gender roles through my participation in Ultimate.

*FINANCIAL BARRIERS*

- The cost of participating in Ultimate (e.g., equipment, travel, fees) limits my ability to fully engage.
- I would play Ultimate more often if financial costs (e.g., registration, travel) were reduced or covered.
- I have missed Ultimate tournaments, practices, or events due to financial reasons.
- Ultimate offers enough financial support (e.g., scholarships, gear lending) to make participation accessible.
- I often worry about being able to afford continued participation in Ultimate.

*AWARENESS BARRIERS*

- Most people I know have never heard of Ultimate.
- It’s difficult to find local opportunities to join an Ultimate team.
- Ultimate is not well represented on social media.
- I discovered Ultimate through word-of-mouth, not through public outreach or advertising.
- USA Ultimate utilizes social media effectively to promote Ultimate.
- Better marketing or social media outreach would help more players get involved in Ultimate.

*Future Participation & Intentions*

1. I intend to continue playing Ultimate in the next 12 months. (Y/N)
   1. [IF NO] Please explain why you do not intend to continue playing Ultimate in the next 12 months.
2. I encourage others to play Ultimate with me. (Y/N)
   1. [IF NO] Please explain why you do not encourage others to play Ultimate.
3. Compared with when I first began playing, I feel more connected now to Ultimate. (Y/N)
   1. [IF NO] Please explain why you do not feel more connected to Ultimate now compared to when you began playing.
4. If I was offered a leadership or coaching role within my Ultimate community I would consider accepting it. (Y/N)
   1. [IF NO] Please explain why you would not consider accepting a leadership or coaching role within your Ultimate community.
5. What are the best things about participating in Ultimate? [OPEN ENDED]
6. What are the main challenges you face when participating in Ultimate? [OPEN ENDED]
7. If you could change on thing about Ultimate to make it more inclusive or supportive, what would you change? [OPEN ENDED]
